# Comparative clinical features of antibiotic-associated Kounis syndrome and non-Kounis allergic coronary events: a disproportionality analysis using U.S. Food and Drug Administration Adverse Event Reporting System

**DOI:** 10.1186/s40780-026-00545-7

**Published:** 2026-02-03

**Authors:** Ichiro Nakakura, Yutaro Mukai, Atsuki Hosoda, Naohiro Ohara, Kaori Yamanishi, Takaya Uno, Satoshi Yokoyama, Kouichi Hosomi, Yoshiko Une

**Affiliations:** 1 Department of Pharmacy, National Cerebral and Cardiovascular Center, 6-1 Kishibe-Shimmachi, Suita, Osaka, 564-8565 Japan; 2 Division of Drug Informatics, School of Pharmacy, Kindai University 3-4-1 Kowakae, Higashiosaka, Osaka , 577-8502 Japan

**Keywords:** Antibiotics, Adverse event, Allergic reaction, Coronary artery disease, Kounis syndrome, Pharmacovigilance database

## Abstract

**Background:**

Kounis syndrome (KS) is an acute coronary syndrome (ACS) triggered by allergic reactions and is frequently associated with antibiotic exposure. However, patient characteristics of antibiotic-associated KS cases are not well described. Some cases with KS-consistent symptoms may instead be reported as antibiotic-associated allergic coronary events (non-KS) without proper diagnosis. Characterizing these non-KS events may improve the accurate diagnosis of KS. This study explores the clinical features of antibiotic-associated KS and non-KS allergic coronary events in the FDA Adverse Event Reporting System (FAERS) and generates hypotheses regarding potential underdiagnosis and improved recognition of KS.

**Methods:**

FAERS reports (September 2012–December 2024) were screened for allergic symptoms (SMQ: Anaphylactic reaction; Hypersensitivity) and ACS (SMQ: Acute myocardial infarction) with antibiotics listed as suspected drugs. After exclusions, 459 cases (KS: 205; non-KS: 254) were included. Extracted variables included demographics, comorbidities, drug use (ATC classification), and outcomes. Logistic regression identified factors independently associated with non-KS classification.

**Results:**

Non-KS group exhibited higher rates of cardiac comorbidities, diabetes, and mortality (all *p* < 0.05). Among suspected antibiotics, penicillins were the only class more frequently reported in KS cases (42% vs. 30%, *p* = 0.010). In the multivariable analysis, factors independently associated with non-KS classification included female sex (odds ratio [OR] = 1.804), fatal outcomes (OR = 4.320), and use of quinolones (OR = 11.108), aminoglycosides (OR = 3.480), renin–angiotensin agents (OR = 2.608), analgesics (OR = 2.055), and polypharmacy (OR = 3.314) (area under the curve = 0.815).

**Conclusions:**

These exploratory findings indicate that non-KS allergic coronary events, characterized by higher comorbidities, mortality, and proportion of cases reporting the use of quinolones or aminoglycosides, may suggest a potential relationship between non-KS coronary events and KS underdiagnosis, warranting further prospective investigation.

**Supplementary Information:**

The online version contains supplementary material available at 10.1186/s40780-026-00545-7.

## Background

Kounis syndrome (KS) is an acute coronary syndrome (ACS) precipitated by mast cell-derived mediators released during allergic reactions, with possible contributions from other immune cells [1, 2]. KS has been reported to cause severe, potentially life-threatening outcomes [1–3]. However, the lack of standardized diagnostic criteria has been linked to its underdiagnosis [1, 2]. It is likely that a subset of patients with ACS and concurrent allergic symptoms are not being recognized as having KS.

Although KS can be triggered by diverse factors, drugs are the most frequent trigger. A systematic review reported that antibiotics accounted for 42.68% of drug-induced cases [2]. Several case reports and pharmacovigilance studies have implicated a broad range of antibiotics as potential KS triggers [2, 4, 5].

We previously examined suspected drugs and patient characteristics in allergy-related coronary events not diagnosed as KS (non-KS) using the Japanese Adverse Drug Event Report (JADER) database [6]. In that study, only a few non-KS cases were associated with suspected antibiotics [6], and the limited sample size precluded a detailed characterization. To our knowledge, the clinical features of suspected antibiotic-associated non-KS cases have not been previously described.

Therefore, as an exploratory, hypothesis-generating analysis, this study aimed to compare the clinical characteristics of antibiotic-associated KS and non-KS allergic coronary events using data from the U.S. Food and Drug Administration (FDA) Adverse Event Reporting System (FAERS). By identifying features that differentiate non-KS cases, this analysis may help generate hypotheses to refine diagnostic approaches and improve clinical recognition of KS.

## Methods

### Data source and case selection

**Fig. 1:**
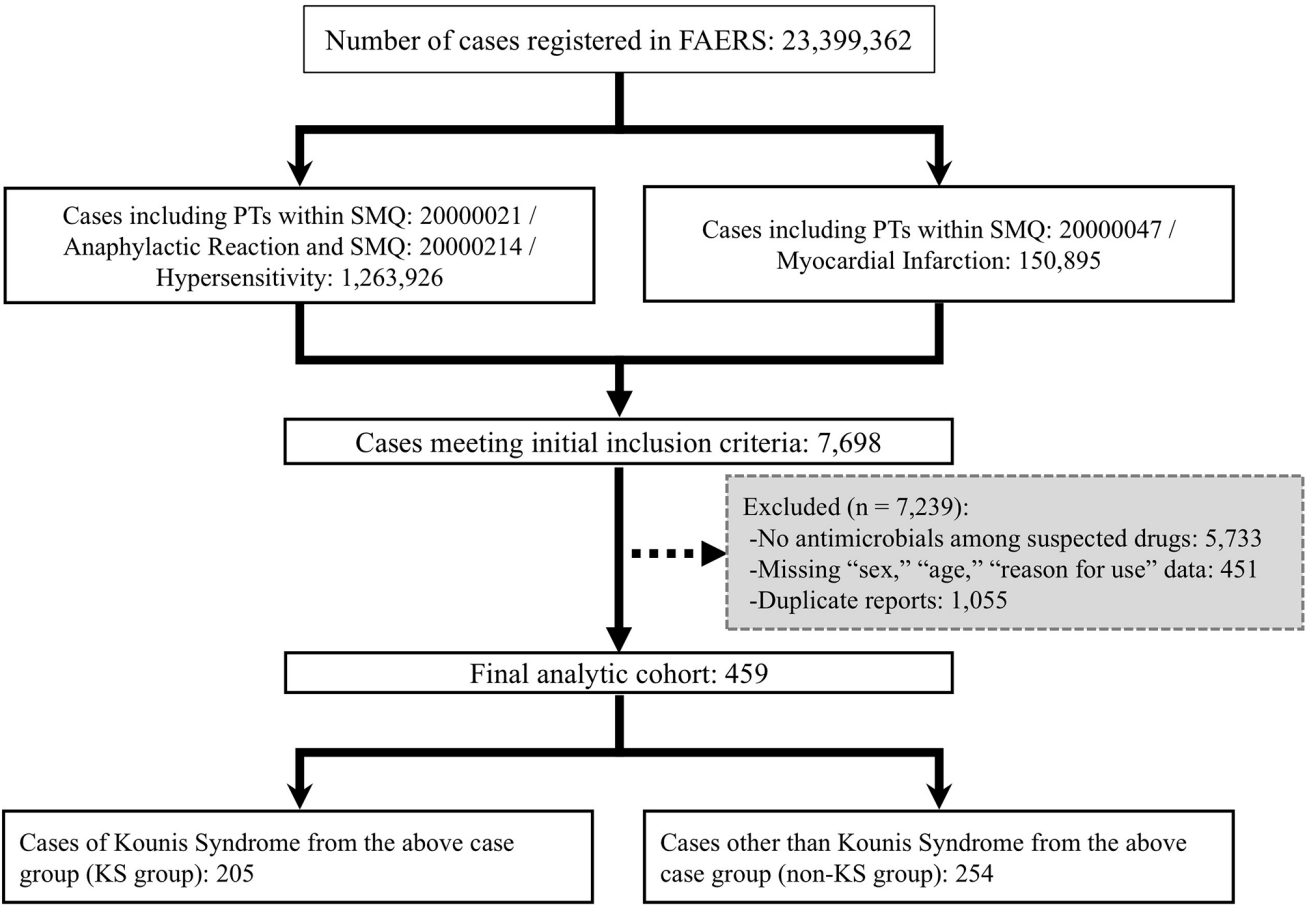
Flowchart of case selection. FAERS: FDA adverse event reporting system, KS: kounis syndrome, PT: preferred Term, SMQ: standardized Medical dictionary for Regulatory Activities queries. Flowchart illustrating the selection process of Kounis syndrome and non-Kounis syndrome cases from the FAERS

The present study utilized data from the FAERS Public Dashboard [7], released by the FDA, covering September 2012 through December 2024. The case selection process is summarized in Fig. 1.

First, we extracted cases from the Reaction dataset that met the following two criteria, based on the Medical Dictionary for Regulatory Activities (MedDRA) version 27.1:

- The presence of at least one Preferred Term (PT) included in the allergy-related Standardised MedDRA Queries (SMQs) “Anaphylactic reaction” (SMQ: 20000021) or “Hypersensitivity” (SMQ: 20000214).
-  The presence of at least one PT included in the SMQ “Acute myocardial infarction” (SMQ: 20000047).

These SMQs were selected because they include the PT “Kounis syndrome” (code: 10,069,167), enabling the identification of cases with allergic symptoms and acute coronary events consistent with KS. This initial selection yielded 7698 cases, of which, 1727 contained the PT “Kounis syndrome.”

Next, from the extracted cases, we excluded reports in which antibiotics were not listed as suspected drugs in the Suspect Product Active Ingredients field. We also excluded cases with missing data for “Reason for Use,” age, sex, and removed duplicate reports. Duplicate cases were identified in accordance with previous approaches [8, 9], defined as records sharing identical values for the following variables: “Literature Reference,” “Sex,” “Event Date,” “Patient Age,” “Country of occurrence,” “Suspect Product Active Ingredients,” and “Outcome.”

Following selection and exclusions, 459 cases were retained for the final analysis. These were classified into two groups based on the following criteria:

KS group: Cases that included the PT “Kounis Syndrome.” 

 Non-KS group: Cases that met the inclusion criteria but did not include the PT “Kounis Syndrome.”

### Variables and definitions

We extracted and analyzed the following variables from the FAERS Public Dashboard: “Suspect Product Active Ingredients,” “Concomitant Product Names,” “Reason for Use,” “Reaction,” “Outcomes,” “Sex,” “Patient Age,” and “Patient Weight”. Drugs listed under “Suspect Product Active Ingredients” and “Concomitant Product Names” were classified using the fifth-level Anatomical Therapeutic Chemical (ATC) classification system [10], excluding unclassifiable agents. Polypharmacy was defined as the use of six or more medications, including suspected and concomitant drugs, consistent with evidence linking this threshold to increased risk of adverse drug reactions [11]. In addition, drugs were classified using the second-level ATC classification system [10], and antibiotics were subclassified according to the third-level ATC code. For each case, the number of distinct antibiotic classes (based on the third-level ATC classification) was counted, and cases involving two or more classes were defined as receiving combination antibiotic therapy. For the final analytical cohort, disease conditions listed in the “Reason for Use” field were extracted based on MedDRA version 27.1, using the definitions in Supplementary Table 1 (See Supplementary Table 1, Additional File 1). These conditions were considered the direct therapeutic targets of either suspected or concomitant drugs and were designated as “diseases under treatment.” As an outcome variable, fatal cases were defined as those in which “Died” was recorded in the “Outcome” field. Since age data were reported in various formats (e.g., years, months, and decades), all values were harmonized to years and summarized in 10‑year intervals for descriptive analyses.

### Data analysis and statistical analysis

Data analysis was conducted in accordance with the READUS-PV (REporting of A Disproportionality analysis for drUg Safety signal detection using spontaneously reported adverse events in PharmacoVigilance) guidelines [12]. The READUS-PV checklist is presented in Supplementary Table 2 (See Supplementary Table 2, Additional File 1). 

To identify factors independently associated with non-KS classification in the final analytical cohort, we performed univariable comparisons and multivariable logistic regression analyses. First, univariable analyses were conducted to compare baseline characteristics, diseases under treatment, and drug use between the KS and non-KS groups. For categorical variables, the chi-square test was used, or Fisher’s exact test when the expected cell counts were < 5. Variables with a p-value < 0.05 were selected for multivariable modeling.

Multivariable logistic regression analysis was then performed using the variables that showed statistically significant differences. Multicollinearity among variables was assessed using the variance inflation factor (VIF), and variables with VIF ≥5 were excluded from the final model. Odds ratios (ORs) and 95% confidence intervals (CIs) were reported, with p-value < 0.05 considered statistically significant. Model goodness-of-fit was evaluated using the Hosmer–Lemeshow test, and discriminatory performance was assessed using the area under the curve (AUC).

To assess the robustness of the findings and evaluate the impact of restricting the primary analysis to reports in which antimicrobials were listed as suspected drugs, we conducted sensitivity analyses using an extended antibiotic cohort (*n* = 472). This cohort comprised the primary cohort (*n* = 459), augmented by 13 reports wherein antimicrobials were recorded only as concomitant drugs. We applied the same data-cleaning procedures and analytical workflow as in the primary analysis.

Statistical analyses were performed using IBM SPSS Statistics version 30 (IBM Corp., Armonk, NY, USA).

### Ethical considerations

As this study utilized anonymized, publicly available data from the FAERS Public Dashboard, ethical review was waived by the Institutional Review Board of the National Cerebral and Cardiovascular Center, in accordance with the Ethical Guidelines for Medical and Health Research Involving Human Subjects. Prior to analysis, we reviewed the limitations of the FAERS Public Dashboard [13] and ensured that the data were used appropriately in line with those limitations.

## Results

### Study population and background characteristics

The final analytical cohort was divided into the KS group (*n* = 205) and the non-KS group (*n* = 254) (Fig. 1).

Background characteristics for both groups are presented in Table 1 and Supplementary Table 3 (See Supplementary Table 3, Additional File 1). The KS group had a higher proportion of men (69%), whereas the non-KS group showed an approximately equal sex distribution (Table 1). In both groups, more than half of the cases were ≥50 years old. However, the KS group included a higher proportion of cases aged 0–39 years compared with the non-KS group (See Supplementary Table 3, Additional File 1). Given that body weight was missing for more than half of the cases in both groups, no comparison between groups was conducted (See Supplementary Table 3, Additional File 1).

**Table 1:** Patient characteristics and fatal outcomes

Characteristic	KS group (N = 205)	Non-KS group (N = 254)	**p-value**^†^
**Sex**			< 0.001
Male	142 (69%)	125 (49%)	
Female	63 (31%)	129 (51%)	
**Diseases under treatment**			
Hypertension	4 (2%)	10 (4%)	0.219
Malignancy	2 (1%)	13 (5%)	0.013
Cardiac disorders	7 (3%)	33 (13%)	< 0.001
Diabetes mellitus	3 (2%)	33 (13%)	< 0.001
Renal failure/impairment	0	1 ( < 1%)	0.369
Allergy conditions	13 (6%)	53 (21%)	< 0.001
Lipid metabolism disorders	3 (2%)	3 (1%)	0.791
**Fatal outcome**	6 (3%)	44 (17%)	< 0.001

Data are presented as n (%)

KS: Kounis syndrome

^†^p-values were calculated using the chi-squared test or Fisher’s exact test, as appropriate. *p* < 0.05 was considered statistically significant

### Diseases under treatment and clinical outcomes

Diseases under treatment and fatal outcomes for the KS and non-KS groups are summarized in Table 1. Compared with the KS group, the non-KS group had significantly more cases with malignant tumors, cardiovascular diseases, diabetes, and allergic conditions (all *p* < 0.05). Additionally, the proportion of fatal outcomes was significantly higher in the non-KS group (KS: 3%, non-KS: 17%, *p* < 0.001).

### Medication use and suspected antibiotics

Table 2 presents a comparison of medication use and suspected antibiotics based on ATC classification. The non-KS group had a significantly higher proportion of cases involving drugs from the following classes: B01 (antithrombotic agents), C01 (cardiac therapy), C07 (beta-blocking agents), C09 (agents acting on the renin–angiotensin system), L01 (antineoplastic agents), L04 (immunosuppressants), M01 (anti-inflammatory agents), and N02 (analgesics) (all *p* < 0.05).

**Table 2:** Medication use and suspected antibiotics

ATC Code/Category	KS group (N = 205)	Non-KS group (N = 254)	**p-value**^**†**^
**Medication use**			
B01 Antithrombotic agents	19 (9%)	93 (37%)	< 0.001
C01 Cardiac therapy	19 (9%)	57 (22%)	< 0.001
C07 Beta blocking agents	7 (3%)	43 (17%)	< 0.001
C09 Agents acting on the renin–angiotensin system	9 (4%)	80 (32%)	< 0.001
L01 Antineoplastic agents	0	45 (18%)	< 0.001
L04 Immunosuppressants	0	51 (20%)	< 0.001
M01 Anti-inflammatory and antirheumatic products	17 (8%)	74 (29%)	< 0.001
N01 Anesthetics	28 (14%)	37 (15%)	0.781
N02 Analgesics	23 (11%)	107 (42%)	< 0.001
V08 Contrast media	0	3 (1%)	0.119
**Suspected Antibiotics**			
J01A TETRACYCLINES	1 ( < 1%)	28 (11%)	< 0.001
J01C BETA-LACTAM ANTIBACTERIALS, PENICILLINS	86 (42%)	77 (30%)	0.010
J01D OTHER BETA-LACTAM ANTIBACTERIALS	65 (32%)	79 (31%)	0.890
J01E SULFONAMIDES AND TRIMETHOPRIM	2 (1%)	44 (17%)	< 0.001
J01F MACROLIDES, LINCOSAMIDES AND STREPTOGRAMINS	21 (10%)	41 (16%)	0.066
J01G AMINOGLYCOSIDE ANTIBACTERIALS	23 (11%)	59 (23%)	0.001
J01M QUINOLONE ANTIBACTERIALS	1 ( < 1%)	39 (15%)	< 0.001
J01X OTHER ANTIBACTERIALS	19 (9%)	27 (10%)	0.629
Polypharmacy*	36 (19%)	158 (62%)	< 0.001
Combination antimicrobials**	10 (5%)	61 (24%)	< 0.001

Data are presented as n (%)

ATC: Anatomical Therapeutic Chemical, KS: Kounis syndrome

*Polypharmacy: use of six or more drugs (suspected and concomitant)

**Combination antimicrobials: two or more antibiotic classes (third-level ATC code)

^**†**^p-values were calculated using the chi-squared test or Fisher’s exact test, as appropriate. *p* < 0.05 was statistically significantMedication use is classified by ATC second-level codes (suspected and concomitant drugs). Suspected antibiotics are classified by ATC third-level codes (suspected drugs only).

Significant differences in antibiotic classes were also observed between the groups (Table 2). In the KS group, the use of J01C (penicillins) was higher compared with the non-KS group (42% vs. 30%, *p* = 0.010). In contrast, the non-KS group had significantly higher use of J01A (tetracyclines), J01E (sulfonamides/trimethoprim), J01M (quinolones), and J01G (aminoglycosides) (all *p* < 0.05). Combination antibiotic therapy was also more common in the non-KS group (24%) than in the KS group (5%) (*p* < 0.001).

### Factors associated with the non-KS group: multivariable analysis

Multivariable logistic regression analysis was conducted using variables with a p-value < 0.05 in the univariate analysis shown in Tables 1 and 2. Multicollinearity was assessed, confirming that all variables had a VIF < 5.

The final multivariable model identified several factors independently associated with non-KS classification (Table 3): female sex (OR = 1.804, 95% CI: 1.141–2.851, *p* = 0.012), fatal outcome (OR = 4.320, 95% CI: 1.674–11.147, *p* = 0.002), use of C09 renin–angiotensin system agents (OR = 2.608, 95% CI: 1.135–5.993, *p* = 0.024), use of N02 analgesics (OR = 2.055, 95% CI: 1.097–3.850, *p* = 0.025), polypharmacy (OR = 3.314, 95% CI: 1.779–5.450, *p* < 0.001), use of J01G aminoglycosides (OR = 3.480, 95% CI: 1.923–6.224, *p* < 0.001), and use of J01M quinolones (OR = 11.108, 95% CI: 1.376–89.705, *p* = 0.024). The final model demonstrated excellent goodness-of-fit (Hosmer–Lemeshow test, *p* = 0.40) and strong discriminatory power (AUC = 0.815).

**Table 3:** Final multivariable logistic regression model for factors associated with the non-KS group

Covariate	Odds Ratio	95% CI	p-value
Female sex	1.804	1.141–2.851	0.012
Fatal outcome	4.320	1.674–11.147	0.002
Use of C09 Renin–Angiotensin System Agents	2.608	1.135–5.993	0.024
Use of N02 Analgesics	2.055	1.097–3.850	0.025
Polypharmacy	3.314	1.779–5.450	< 0.001
Use of J01G Aminoglycoside Antibacterials	3.480	1.923–6.224	< 0.001
Use of J01M Quinolone Antibacterials	11.108	1.376–89.705	0.024

Model fit statistics: Hosmer–Lemeshow test, *p* = 0.40; area under the curve (AUC) = 0.815.CI: confidence interval, KS: Kounis syndrome

In the sensitivity analysis using the extended antibiotic cohort(*n* = 472), the multivariable logistic regression model demonstrated good discriminatory performance(AUC = 0.813). Factors independently associated with non-KS classification included fatal outcome(OR = 4.992, 95% CI: 1.937–12.864), use of C09 renin–angiotensin system agents (OR = 3.331, 95% CI: 1.474–7.528), use of L04 immunosuppressants(OR = 19.177, 95% CI: 4.409–83.411), polypharmacy(OR = 2.260, 95% CI: 1.341–3.810), and use of J01G aminoglycosides (OR = 3.508, 95% CI: 1.960–6.276)and J01M quinolones (OR = 20.960, 95% CI: 2.694–163.087)(Supplementary Tables 4–6).

## Discussion

To our knowledge, this exploratory, hypothesis-generating study is the first to compare clinical characteristics of antibiotic-associated KS and non-KS events using FAERS data. The non-KS group differed from the KS group across several domains, including patient demographics, comorbidities, and antibiotic classes used.

Because KS is a rare adverse event [1, 3], large-scale spontaneous reporting systems, such as FAERS, can contribute valuably to early signal detection beyond the scope of clinical trials. Although these databases are not suitable for estimating incidence or establishing causality, we applied multivariable logistic regression to examine independent associations with non-KS classification after adjustment for multiple covariates. Nevertheless, this analysis was conducted solely for exploratory, hypothesis-generating purposes. Accordingly, in line with the FAERS Public Dashboard FAQ [13] and READUS-PV reporting recommendations [12], our FAERS-derived findings should not be interpreted as definitive predictors or risks but as testable hypotheses that warrant validation in future clinical datasets or pharmacoepidemiologic studies. The multivariable analysis identified factors associated with non-KS classification, such as fatal outcomes, use of specific drugs (e.g., renin–angiotensin agents and analgesics), antibiotics (e.g., quinolones and aminoglycosides), and polypharmacy. These observations help generate hypotheses relevant to refining clinical recognition and diagnostic algorithms of KS.

The higher mortality observed in the non-KS group compared with the KS group is notable and suggests more severe underlying clinical conditions. This observation is consistent with our previous report [6]. In univariate analysis, non-KS cases displayed a higher burden of comorbidities (e.g., cardiovascular disease, diabetes), increased rates of polypharmacy, and greater use of medications such as beta-blockers (C07), renin–angiotensin agents (C09), and anti-inflammatory agents (M01).

Several mechanisms may contribute to the elevated mortality in the non-KS group: (1) worsened ACS prognosis due to heart disease and diabetes [14]; (2) pre-existing cardiovascular disease increases the risk of severe or fatal drug-induced anaphylaxis [15]; and (3) beta-blockers, angiotensin converting enzyme inhibitors/angiotensin II receptor blockers, and anti-inflammatory agents may impair compensatory responses or reduce the efficacy of epinephrine [16]. This combination of factors may help explain the elevated mortality in the non-KS group, consistent with our prior JADER-based analysis [6].

In addition to severity indicators, patient demographics also differed between groups. KS is more frequently reported in men [2], consistent with our KS group. In contrast, the non-KS group showed an approximately equal sex distribution, and multivariable analysis identified female sex as being independently associated with non-KS classification (OR = 1.804, 95% CI: 1.141–2.851, *p* = 0.012). This may suggest that the non-KS group included a relatively higher proportion of female sex, potentially a distinct clinical profile associated with increased comorbidities and mortality.

Another noteworthy observation was the high prevalence of multiple comorbidities—including malignancies, cardiovascular disease, and diabetes—in the non-KS group, many of whom were also affected by polypharmacy, suggesting a more complex clinical background. In such patients, acute clinical presentations may be masked or mimicked by pre-existing symptoms, posing challenges to timely and accurate diagnosis [17]. Polypharmacy further complicates diagnosis by making it more difficult to identify the specific drug responsible for adverse reactions [18]. These findings suggest that in patients with multiple comorbidities and polypharmacy, allergy-associated coronary events may be misattributed to underlying disease or unrelated adverse effects—leading to missed or delayed diagnoses of KS. In the absence of standardized diagnostic criteria, these complex presentations may play a substantial role in KS underdiagnosis and the poor outcomes observed.

Notably, the independent association between use of N02 analgesics and the non-KS group suggests a potential link between analgesic use and the clinical characteristics of this population. The N02 analgesic category includes opioid analgesics such as morphine, as well as antipyretic analgesics such as aspirin and metamizole. Morphine is known to trigger anaphylactoid reactions through non-specific mast cell degranulation [19], and KS has been reported in association with analgesics such as morphine and aspirin [20, 21]. However, the direct induction of ACSs by these agents is not commonly reported. This apparent inconsistency is likely attributable to confounding factors, particularly “confounding by indication” [22]. As previously noted, the non-KS group exhibited a higher prevalence of severe underlying conditions, including malignancies, many of which require analgesic therapy. Therefore, the use of analgesics in the non-KS group may be less indicative of a direct causal relationship with allergy-related coronary events and more reflective of severe, complex comorbidities.

Another important finding was the notable difference in antibiotic classes between the KS and non-KS groups. Consistent with previous reports [2, 4, 5], the KS group was predominantly associated with penicillin-class antibiotics. In contrast, the use of quinolones (OR = 11.108) and aminoglycosides (OR = 3.480) were independently associated with the non-KS group. Penicillins and quinolones are well-recognized causes of drug-induced anaphylaxis [23], whereas aminoglycoside-induced immediate hypersensitivity reactions are rarely reported [24]. This disparity, despite known differences in allergenic potential, suggests that factors beyond incidence of hypersensitivity may underlie the observed distribution.

Mechanistic differences between antibiotic classes may be relevant. Hypersensitivity to penicillins typically involves classical IgE-mediated mast cell activation, the hallmark of immediate (Type I) hypersensitivity [25]. In this pathway, drug-specific antibodies formed during sensitization bind to mast cells. Upon re-exposure, mast cell degranulation occurs, releasing inflammatory mediators such as histamine and producing the clinical manifestations of a Type I hypersensitivity reaction. The well-defined and rapid-onset nature of this immediate-type reaction likely facilitates recognition of its link to acute events like KS. In contrast, quinolone antibiotics are known to trigger IgE-mediated and non-IgE-mediated hypersensitivity reactions [25]. One notable non-IgE-mediated mechanism involves direct activation of Mas-related G protein-coupled receptor X2 (MRGPRX2), abundantly expressed on cutaneous mast cells, which induces IgE-independent degranulation (formerly termed pseudo-allergy or anaphylactoid reactions) [25]. These reactions may present with urticaria, angioedema, and anaphylaxis, mimicking immediate-type hypersensitivity [25]. IgE-mediated immediate reactions to aminoglycosides have also been reported, but they remain exceptionally rare and largely confined to isolated case reports [24]. Most aminoglycoside hypersensitivity reactions are instead T-cell–mediated Type IV (delayed-type) responses [24]. Thus, the antibiotic classes implicated in the KS and non-KS groups appear to act through divergent immunological pathways. Quinolones and aminoglycosides, which predominated in the non-KS group, may trigger non-IgE-mediated or delayed-type hypersensitivity, unlike the classical penicillin-induced pathway. This mechanistic diversity, particularly differences in symptom onset, may complicate clinical assessment and may contribute to the underdiagnosis of KS in patients exposed to these agents.

The use of combination antibiotic therapy may further obscure diagnosis. The non-KS group showed a significantly higher proportion of combination therapy involving multiple antibiotic classes compared to the KS group (KS group: 5%, non-KS group: 24%, *p* < 0.001, Table 2). Combination regimens are often used for severe infections, where poor systemic condition and multiple potential causative agents may hinder identification of the responsible drug, leading to missed or delayed KS diagnoses.

Although our previous study [6] using the JADER database compared KS and non-KS groups, the limited number of antibiotic-associated non-KS cases precluded detailed characterization of this subgroup. By leveraging the larger, international sourced FAERS database, the present study identified novel antibiotic-specific patterns not apparent in JADER, specifically, the significant association of penicillins with the KS group, in contrast to the strong association of quinolones and aminoglycosides with the non-KS group. This finding reflects the diverse and international characteristics of the FAERS database, which likely revealed associations not readily apparent in the more homogeneous, Japan-specific JADER database.

In the sensitivity analysis using the extended cohort, several adjusted associations exhibited similar directions to those in the primary analysis (fatal outcome, use of renin–angiotensin system agents, polypharmacy, and use of aminoglycosides and quinolones). Conversely, the adjusted associations with female sex and analgesic use observed in the primary analysis were attenuated, whereas immunosuppressant use emerged as an associated factor in the extended cohort. These differences indicate that some observed associations may be sensitive to cohort definition, residual confounding, or both, underscoring the hypothesis-generating nature of these findings.

Clinicians may consider maintaining heightened awareness of KS in patients receiving quinolones or aminoglycosides, even when the clinical presentations are atypical. As these observations are exploratory, validation in independent clinical datasets with verified diagnoses is warranted. Nevertheless, some limitations should be acknowledged when interpreting these results.

### Study limitations

First, as a spontaneous reporting system, FAERS is inherently subject to biases such as underreporting and selective reporting. Second, the diagnostic accuracy of individual cases could not be verified because of the lack of detailed clinical information (e.g., electrocardiograms, coronary angiography, or allergy test results), which limits the ability to establish causality. Third, selection bias may have been introduced by the exclusion of cases with missing key variables, specifically sex, age, and reason for use, as shown in Fig. 1. Although necessary for analytical consistency, these exclusions may have affected the representativeness of the final cohort. Fourth, because this study relied on the FAERS Public Dashboard, systematic identification of drugs coded as “interacting” (role code I) in the underlying FAERS DRUG dataset was not possible. Consequently, the potential contribution of interacting co-medications to the reported events or the observed comparisons could not be evaluated. Fifth, although a previous systematic review reported that antibiotics accounted for 42.68% of drug-induced KS cases, this figure was based on case reports and observational studies that are subject to reporting bias and lack standardized data-cleaning procedures. In contrast, our FAERS-based analysis identified only 459 antibiotic-associated reports among 7698 suspected-drug reports (approximately 6%). Sixth, the sensitivity analysis incorporated only additional 13 reports wherein antibiotics were recorded exclusively as concomitant drugs. This modest increase may have reduced statistical power to detect subtle differences; therefore, residual selection bias related to cohort definition cannot be fully ruled out.

This discrepancy may reflect differences in data sources, extraction criteria, and methodological rigor, including the removal of duplicate reports. These limitations, while inherent to spontaneous reporting systems, underscore that FAERS is best suited for hypothesis generation rather than causal inference. To address these limitations and validate our findings, future research should include retrospective analyses using electronic health records or prospective observational studies with richer clinical data.

## Conclusions

This study provides the first comparative analysis of antibiotic-associated KS and non-KS coronary events using the FAERS database. The non-KS group was characterized by a higher burden of comorbidities, increased mortality, and a higher frequency of quinolone and aminoglycoside use. These findings suggest a potential relationship between non-KS coronary events and KS underdiagnosis. Greater awareness of the non-KS profile is essential to enhance diagnostic accuracy and improve patient outcomes.

## Electronic supplementary material

Below is the link to the electronic supplementary material.

Supplementary material 1

## Data Availability

The data used in this study were obtained from the publicly available FAERS Public Dashboard provided by the U.S. Food and Drug Administration (https://www.fda.gov/drugs/fdas-adverse-event-reporting-system-faers/fda-adverse-event-reporting-system-faers-public-dashboard). Drug classification was performed using the WHO Anatomical Therapeutic Chemical codes (https://www.whocc.no/atc_ddd_index/). Various diseases and adverse events were categorized using MedDRA coding systems, including Standardised MedDRA Queries (SMQs), System Organ Class (SOC), High Level Group Term (HLGT), High Level Term (HLT), and Preferred Terms (PTs).

## Abbreviations

ACS: Acute Coronary Syndrome
ATC: Anatomical Therapeutic Chemical
AUC: Area Under the Curve
CI: Confidence Intervals
FAERS: FDA Adverse Event Reporting System
FDA: Food and Drug Administration
JADER: Japanese Adverse Drug Event Report
KS: Kounis Syndrome
MRGPRX2: Mas-related G protein-coupled receptor X2
MedDRA: Medical Dictionary for Regulatory Activities
ORs: Odds Ratios
PT: Preferred Term
READUS-PV: REporting of A Disproportionality analysis for drUg Safety signal detection using spontaneously reported adverse events in PharmacoVigilance
SMQs: Standardised MedDRA Queries
VIF: Variance Inflation Factor

## References

[1] Kounis NG. Kounis syndrome: an update on epidemiology, pathogenesis, diagnosis and therapeutic management. Clin Chem Lab Med. 2016;54:1545–59. 10.1515/cclm-2016-0010. 26966931 10.1515/cclm-2016-0010

[2] Cahuapaza-Gutierrez NL, Calderon-Hernandez CC, Chambergo-Michilot D, De Arruda-Chaves E, Zamora A, Runzer-Colmenares FM. Clinical characteristics, management, diagnostic findings, and various etiologies of patients with Kounis syndrome. A systematic review. Int J Cardiol. 2025;418:132606. 10.1016/j.ijcard.2024.132606. 39362367 10.1016/j.ijcard.2024.132606

[3] Desai R, Parekh T, Patel U, et al. Epidemiology of acute coronary syndrome co-existent with allergic/hypersensitivity/anaphylactic reactions (Kounis syndrome) in the United States: a nationwide inpatient analysis. Int J Cardiol. 2019;292:35–38. 10.1016/j.ijcard.2019.06.002. 31204069 10.1016/j.ijcard.2019.06.002

[4] Renda F, Marotta E, Landoni G, Belletti A, Cuconato V, Pani L. Kounis syndrome due to antibiotics: a global overview from pharmacovigilance databases. Int J Cardiol. 2016;224:406–11. 10.1016/j.ijcard.2016.09.066. 27684599 10.1016/j.ijcard.2016.09.066

[5] Orion K, Luck J, Kullak-Ublick GA, Weiler S. Kounis syndrome: a retrospective analysis of individual case safety reports from the international WHO database in pharmacovigilance. Int J Clin Pharmacol Ther. 2019;57:240–48. 10.5414/cp203344. 30900982 10.5414/CP203344

[6] Nakakura I, Mukai Y, Hosoda A, et al. Association between Kounis syndrome and Allergy-associated coronary events: a Disproportionality analysis using the Japanese adverse drug event report database. Cureus. 2025;17:e90613. 10.7759/cureus.90613. 40979021 10.7759/cureus.90613 PMC12449037

[7] FDA adverse event reporting system (FAERS) Public Dashboard. Available from: https://www.fda.gov/drugs/fdas-adverse-event-reporting-system-faers/fda-adverse-event-reporting-system-faers-public-dashboard. (accessed 3 January 2025.

[8] Norén GN, Orre R, Bate A, et al. Duplicate detection in adverse drug reaction surveillance. Data Min Knowl Disc. 2007;14:305–28. 10.1007/s10618-006-0052-8.

[9] Han W, Morris R, Bu K, Zhu T, Huang H, Cheng F. Analysis of literature-derived duplicate records in the FDA adverse event reporting system (FAERS) database. Can J Physiol Pharmacol. 2025;103:56–69. 10.1139/cjpp-2024-0078. 39620731 10.1139/cjpp-2024-0078

[10] World Health Organization. ATC/DDD Index. 2025]. Available from: https://www.whocc.no/atc_ddd_index/. (accessed 5 March 2025).

[11] Kojima T, Akishita M, Kameyama Y, et al. High risk of adverse drug reactions in elderly patients taking six or more drugs: analysis of inpatient database. Geriatr Gerontol Int. 2012;12:761–62. 10.1111/j.1447-0594.2012.00868.x. 22998384 10.1111/j.1447-0594.2012.00868.x

[12] Fusaroli M, Salvo F, Begaud B, et al. The REporting of a Disproportionality analysis for DrUg Safety signal detection using individual case Safety reports in PharmacoVigilance (READUS-PV): explanation and elaboration. Drug Saf. 2024;47:585–99. 10.1007/s40264-024-01423-7. 38713347 10.1007/s40264-024-01423-7 PMC11116264

[13] FAERS Public Dashboard - FAQ. Available from: https://fis.fda.gov/extensions/FPD-FAQ/FPD-FAQ.html. (accessed 3 January 2025).

[14] Hokimoto S, Kaikita K, Yasuda S, et al. JCS/CVIT/JCC, 2023 guideline focused update on diagnosis and treatment of vasospastic Angina (coronary spastic Angina) and coronary microvascular dysfunction. Circ J. 2023;87:879–936. 10.1253/circj.cj-22-0779. 36908169 10.1253/circj.CJ-22-0779

[15] Turner PJ, Jerschow E, Umasunthar T, Lin R, Campbell DE, Boyle RJ. Fatal anaphylaxis: mortality rate and risk factors. J Allergy Clin Immunol Pract. 2017;5:1169–78. 10.1016/j.jaip.2017.06.031. 28888247 10.1016/j.jaip.2017.06.031 PMC5589409

[16] Beyaz Ş, Gelincik A. Anaphylaxis in risky populations. Curr Pharm Des. 2023;29:224–38. 10.2174/1381612829666221207105214. 36503444 10.2174/1381612829666221207105214

[17] Valderas JM, Starfield B, Sibbald B, Salisbury C, Roland M. Defining comorbidity: implications for understanding health and health services. Ann Fam Med. 2009;7:357–63. 10.1370/afm.983. 19597174 10.1370/afm.983 PMC2713155

[18] de Anda-Jáuregui G, Guo K, Hur J. Network-based assessment of adverse drug reaction risk in polypharmacy using High-throughput screening data. Int J Mol Sci. 2019;20:386. 10.3390/ijms20020386. 30658437 10.3390/ijms20020386 PMC6358820

[19] Rico Cepeda P, Palencia Herrejón, Rodríguez Aguirregabiria. Síndrome de Kounis [Kounis syndrome]. Med Intensiva. 2012;36:358–64. 10.1016/j.medin.2011.10.008. 22154226 10.1016/j.medin.2011.10.008

[20] Akgullu C, Eryilmaz U, Gungor H, et al. Myocardial infarction secondary to morphine-induced Kounis syndrome. Herz. 2014;39:874–76. 10.1007/s00059-013-3919-7. 23907697 10.1007/s00059-013-3919-7

[21] Wang C, Fang W, Song L, et al. Analysis of clinical features of non-steroidal anti-inflammatory drugs induced Kounis syndrome. Front Cardiovasc Med. 2022;9:901522. 10.3389/fcvm.2022.901522. 35898282 10.3389/fcvm.2022.901522 PMC9309368

[22] Collaboration C, Aronson JK, Bankhead C, Mahtani KR, Nunan D. Confounding by indication. Catalogue Of Biases. 2018. https://catalogofbias.org/biases/confounding-by-indication. (accessed 3 September 2025).

[23] Regateiro FS, Marques ML, Gomes ER. Drug-induced anaphylaxis: an update on epidemiology and risk factors. Int Arch Allergy Immunol. 2020;181:481–87. 10.1159/000507445. 32396909 10.1159/000507445

[24] Dilley M, Geng B. Immediate and delayed hypersensitivity reactions to antibiotics: aminoglycosides, clindamycin, Linezolid, and metronidazole. Clin Rev Allergy Immunol. 2022;62:463–75. 10.1007/s12016-021-08878-x. 34910281 10.1007/s12016-021-08878-x PMC9156451

[25] Kolkhir P, Ali H, Babina M, et al. MRGPRX2 in drug allergy: what we know and what we do not know. J Allergy Clin Immunol. 2023;151:410–12. 10.1016/j.jaci.2022.09.004. 36089079 10.1016/j.jaci.2022.09.004 PMC9905269

