# Supplementary material for: Comparative clinical features of antibiotic-associated Kounis syndrome and non-Kounis allergic coronary events: a disproportionality analysis using U.S. Food and Drug Administration Adverse Event Reporting System
Source: J Pharm Health Care Sci. 2026 Feb 3;12:28. doi: 10.1186/s40780-026-00545-7 (PMC12958633; doi:10.1186/s40780-026-00545-7)
Supplement: Supplementary file 1 — Supplementary material 1 [file 40780_2026_545_MOESM1_ESM.docx]

**Additional File 1**

**Supplementary Table 1.** **Target diseases based on reason for use in FAERS (MedDRA classification)**

| Target Diseases | Definitions |
| --- | --- |
| Hypertension | Cases with PTs: Hypertension (10020772) and/or Essential hypertension (10015488) |
| Malignancy | Cases with PTs included in the SOC: Neoplasms benign, malignant and unspecified (incl cysts and polyps) (10029104), and also included under the HLGT: Malignant neoplasms (10010331). |
| Cardiac Disorder | Cases with PTs included in the SOC: Cardiac disorders (10007541). |
| Diabetes | Cases with PTs included in the HLT: Diabetes mellitus (including subtypes) (10012602). |
| Renal Impairment | Cases with any PT included in the HLT: Renal failure and impairment (10038443). |
| Allergy | Cases with PTs included in the SOC: Immune system disorders (10021428), under the HLGT: Allergic conditions (10001708). |
| Lipid Metabolism Disorder | Cases with PTs included in the SOC: Metabolism and nutrition disorders (10027433), under the HLGT: Lipid metabolism disorders (10013317). |

FAERS: FDA Adverse Event Reporting System, HLGT: High Level Group Term, HLT: High Level Term, MedDRA: Medical Dictionary for Regulatory Activities, PT: Preferred Term, PTs: Preferred Terms, SOC: System Organ Class.

Disease definitions are based on MedDRA version 27.1 and derived from the “Reason for Use” field in the FAERS database.

**Supplementary Table 2. The READUS-PV checklist**

| **Section and topic** | **Item #** | **Checklist item** | **Location where item is reported** |
| --- | --- | --- | --- |
| **Title** |  |  |  |
|  | ***1a*** | ***If disproportionality analyses are a prominent component of the published study, the study should be identified as a “disproportionality analysis”. The type of data and name of the database(s) should be specified.*** | Title page, Abstract |
|  | ***1b*** | ***Report the name of adverse event(s) and/or drug(s) under study, when applicable.*** | Title page, Abstract, Keywords |
| **Introduction** |  |  |  |
| **Background** | ***2a*** | ***Describe the drug(s) and its utilization, the nature of the adverse event(s) under study and its frequency, and the existing knowledge on the drug-event combination.*** | Introduction, Discussion |
|  | ***2b*** | ***Specify the rationale for performing the analysis, e.g., as part of routine pharmacovigilance, to investigate an overall safety profile, or to assess a pre-specified hypothesis.*** | Introduction |
|  | ***2c*** | ***Explain why ICSR databases and disproportionality analysis are suitable to fill the knowledge gap.*** | Introduction, Method |
| **Objectives** | ***3*** | ***State specific objectives, identifying the adverse event(s), the drug(s), and the reference group, including any pre-specified hypothesis, if applicable.*** | Abstract, Introduction |
| **Methods** |  |  |  |
| **Study design** | ***4a*** | ***Identify the study (i.e., “disproportionality analysis”) and the type of data used (e.g., “individual case safety reports”).*** | Methods; Data Analysis and Statistical Analysis |
|  | ***4b*** | ***Provide an outline of the entire study design, including primary and sensitivity analyses performed, and other designs such as case-by-case analysis or literature review.*** | Methods; Data Analysis and Statistical Analysis. Figure 1 |
| **Data description, access, and pre-processing** | ***5a*** | ***Specify the name of the database(s), the database(s) custodian, and the coverage. Specify the type/number of drugs included within the database and the thesaurus, taxonomies, or ontologies used for coding drugs and events.*** | Methods |
|  | ***5b*** | ***Specify the extraction dates and describe and justify all choices used for data pre-processing, including any data transformation or exclusion, if appropriate.*** | Methods, Figure 1 |
| **Variables definition** | ***6a*** | ***Describe the study population, including any restriction.*** | Methods; Variables and Definitions |
|  | ***6b*** | ***Describe the nature and the meaning of key variables assessed in the work.*** | Methods; Variables and Definitions |
|  | ***6c*** | ***Specify and justify any grouping of drugs or events. For drugs, specify and justify whether active ingredients/trade names/salts were considered and/or the selected role.*** | Methods; Variables and Definitions |
|  | ***6d*** | ***Describe any additional data source used, the type of data, and how they interact with ICSRs.*** | Not applicable (no additional data sources reported) |
| **Statistical methods** | ***7a*** | ***Present any descriptive analysis performed, specifying variables investigated, statistical tests, and significance thresholds.*** | Methods; Data Analysis and Statistical Analysis, Tables 1-2 |
|  | ***7b*** | ***Describe the measure(s) selected for the disproportionality analysis including any threshold used to identify signals of disproportionate reporting. Explain the reason for this choice if applicable.*** | Methods; Data Analysis and Statistical Analysis |
|  | ***7c*** | ***Clearly describe any sensitivity analysis and any tool to control confounding, including any restriction, subgroup, stratification, adjustment, or interaction.*** | Methods; Data Analysis and Statistical Analysis |
|  | ***7d*** | ***Specify the variables and methods used for the case-by-case analysis, including any algorithm or criteria used to assess causality, if performed.*** | Not applicable (no case-by-case analysis reported) |
|  | ***7e*** | ***Specify any statistical methods used for other data sources.*** | Not applicable (no other data sources reported) |
| **Results** |  |  |  |
| **Participants** | ***8a*** | ***Specify the number of individual case safety reports included at each stage, including reasons for exclusion.*** | Methods, Figure 1 |
|  | ***8b*** | ***Provide key demographic and clinical characteristics of cases, if possible comparing cases with any appropriate reference group.*** | Results, Tables 1-3, Supplementary Table 3 |
| **Disproportionality analysis** | ***9*** | ***Present all results including confidence intervals. Present also results of sensitivity analyses, if performed.*** | Results, Tables 1-3 Additional File: Supplementary Table 4-6 |
| **Case-by-case analysis** | ***10*** | ***Present the case-by-case analysis of key variables. Present the causality assessment, if applicable.*** | Not applicable (no case-by-case analysis reported) |
| **Discussion** |  |  |  |
| **Key results** | ***11*** | ***Discuss key results with reference to study objectives and contextualize them within the current literature and other consulted sources. Clearly discriminate between expected reactions and emerging safety signals.*** | Discussion |
| **External validity** | ***12a*** | ***Discuss the external validity of the results to the general population.*** | Discussion |
|  | ***12b*** | ***Discuss the potential relevance of results in clinical practice*** | Discussion, Conclusions |
|  | ***12c*** | ***Propose further study designs if applicable*** | Discussion; Limitations of This Study |
| **Limitations** | ***13*** | ***Present general limitations, making clear that disproportionality analysis alone cannot prove causation or measure incidence, and specific limitations, including confounding and reporting bias and efforts to mitigate them.*** | Discussion; Limitations of This Study |
| **Declarations** |  |  |  |
|  | ***14a*** | ***Provide the source of funding/sponsorship and the role of the funders/sponsors for the present study and for any original study on which the present article is based.*** | Declarations; Funding |
|  | ***14b*** | ***Clearly identify potential commercial and intellectual conflicts of interest (e.g., link to any drug/event investigated, whether financial, legal action, or software used).*** | Declarations; Conflict of interest |
|  | ***14c*** | ***Declare any institutional approval needed or granted in the investigation.*** | Methods; Ethical Considerations |
|  | ***14d*** | ***Include a statement on data availability, code availability (including the version of the statistical software used), and protocol registration.*** | Declarations; Availability of data and materials |

***Note: The READUS-PV checklist was obtained from https://readus-statement.org/readus-statement/. (accessed 30 August 2025).**

READUS-PV: REporting of A Disproportionality analysis for drUg Safety signal detection using spontaneously reported adverse events in PharmacoVigilance

**Supplementary Table 3. Patient characteristics by age and body weight**

|  | **KS group (N=205)** | **non-KS group (N=254)** |
| --- | --- | --- |
| **Age (years)** |  |  |
| 0-9 | 0 | 1 |
| 10-19 | 6 | 3 |
| 20-29 | 14 | 5 |
| 30-39 | 27 | 6 |
| 40-49 | 15 | 27 |
| 50-59 | 51 | 90 |
| 60-69 | 40 | 60 |
| 70-79 | 35 | 41 |
| 80-89 | 14 | 20 |
| ≥90 | 3 | 1 |
| **Body weight (kg)** |  |  |
| ≤39 | 0 | 1 |
| 40-49 | 1 | 5 |
| 50-59 | 2 | 10 |
| 60-69 | 8 | 39 |
| 70-79 | 4 | 17 |
| 80-89 | 2 | 15 |
| 90-99 | 1 | 11 |
| ≥100 | 1 | 19 |
| Not Available | 186 | 137 |

KS: Kounis syndrome 
The chi-squared test was not performed for age categories due to small numbers in some cells, and for weight categories due to a large proportion of missing data.

**Supplementary Table 4. Patient characteristics and fatal outcomes of extended cohort (n = 472)**

| **​Characteristic** | **KS group (N=216)** | **Non-KS group (N=256)** | **p-value**^†^ |
| --- | --- | --- | --- |
| **Sex** |  |  | <0.001 |
| Male | 145 (67%) | 125 (49%) |  |
| Female | 71 (33%) | 131 (51%) |  |
| **Diseases under treatment** |  |  |  |
| Hypertension​ | 4 (2%) | 10 (4%) | 0.190 |
| Malignancy​ | 2 (1%) | 14 (5%) | 0.007 |
| Cardiac disorder​s | 8 (4%) | 33 (13%) | <0.001 |
| Diabetes​ mellitus | 3 (1%) | 33 (13%) | <0.001 |
| Renal failure/impairment | 0 | 1 (<1%) | 0.358 |
| Allergy​ conditions | 13 (6%) | 53 (21%) | <0.001 |
| Lipid metabolism disorders | 3 (1%) | 3 (1%) | 0.834 |
| **Fatal outcome**​ | 6 (3%) | 46 (18%) | <0.001 |

**Data are presented as n (%).**

KS: Kounis syndrome 
† p-values were calculated using the chi-squared test or Fisher's exact test, as appropriate. p<0.05 was considered statistically significant.

**Supplementary Table 5. Medication use and suspected antibiotics of extended cohort (n = 472)**

| **ATC Code / Category** | **KS group (N=216)** | **Non-KS group (N=256)** | **p-value​^†^** |
| --- | --- | --- | --- |
| **Medication use** |  |  |  |
| B01 Antithrombotic agents​ | 23 (11%) | 94 (37%) | <0.001 |
| C01 Cardiac therapy​ | 23 (11%) | 57 (22%) | <0.001 |
| C07 Beta blocking agents​ | 9 (4%) | 43 (17%) | <0.001 |
| C09 Agents acting on the renin–angiotensin system​ | 10 (5%) | 80 (31%) | <0.001 |
| L01 Antineoplastic agents​ | 0 | 47 (18%) | <0.001 |
| L04 Immunosuppressants​ | 2 (1%) | 51 (20%) | <0.001 |
| M01 Anti-inflammatory and antirheumatic products​ | 21 (10%) | 74 (29%) | <0.001 |
| N01 Anesthetics​ | 33 (15%) | 37 (15%) | 0.802 |
| N02 Analgesics​ | 29 (13%) | 108 (42%) | <0.001 |
| V08 Contrast media​ | 0 | 3 (1%) | 0.111 |
| **Suspected Antibiotics** |  |  |  |
| J01A TETRACYCLINES | 2 (1%) | 29 (11%) | <0.001 |
| J01C BETA-LACTAM ANTIBACTERIALS, PENICILLINS | 87 (40%) | 77 (30%) | 0.027 |
| J01D OTHER BETA-LACTAM ANTIBACTERIALS | 70 (32%) | 79 (31%) | 0.710 |
| J01E SULFONAMIDES AND TRIMETHOPRIM | 4 (2%) | 45 (18%) | <0.001 |
| J01F MACROLIDES, LINCOSAMIDES AND STREPTOGRAMINS | 23 (11%) | 41 (16%) | 0.090 |
| J01G AMINOGLYCOSIDE ANTIBACTERIALS | 23 (11%) | 59 (23%) | <0.001 |
| J01M QUINOLONE ANTIBACTERIALS | 1 (<1%) | 39 (15%) | <0.001 |
| J01X OTHER ANTIBACTERIALS | 20 (9%) | 27 (11%) | 0.629 |
| **Polypharmacy*** | 45 (21%) | 160 (63%) | <0.001 |
| **Combination antimicrobials**** | 11 (5%) | 61 (24%) | <0.001 |

Data are presented as n (%).

ATC: Anatomical Therapeutic Chemical, KS: Kounis syndrome.

*Polypharmacy: use of six or more drugs (suspected and concomitant).

**Combination antimicrobials: two or more antibiotic classes **(**third-level ATC code).

† p-values were calculated using the chi-squared test or Fisher’s exact test, as appropriate. p<0.05 was statistically significant.

Medication use is classified by ATC second-level codes (suspected and concomitant drugs). Suspected antibiotics are classified by ATC third-level codes (suspected drugs only).

**Supplementary Table 6. Multivariable logistic regression for factors associated with the non‑KS group in the extended antibiotic cohort (n = 472)**

| **Covariate** | **Odds Ratio** | **95% CI** | **p-value** |
| --- | --- | --- | --- |
| Fatal outcome | 4.992 | 1.937-12.864 | <0.001 |
| Use of C09 Renin–Angiotensin System Agents | 3.331 | 1.474-7.528 | 0.010 |
| Use of L04 Immunosuppressants | 19.177 | 4.409-83.411 | <0.001 |
| Polypharmacy | 2.260 | 1.341-3.810 | 0.002 |
| Use of J01G Aminoglycoside Antibacterials | 3.508 | 1.960-6.276 | <0.001 |
| Use of J01M Quinolone Antibacterials | 20.960 | 2.694-163.087 | 0.004 |

Model fit statistics: Hosmer–Lemeshow test, p=0.475; area under the curve (AUC)=0.813.

CI: confidence interval, KS: Kounis syndrome.
